# Artificial Intelligence (AI) tools to investigate virulence and evaluate the infectivity of Mpox

**DOI:** 10.17179/excli2025-8129

**Published:** 2025-03-06

**Authors:** Rajiv Gandhi Gopalsamy, Athesh Kumaraswamy, Marina dos Santos Barreto, Ronaldy Santana Santos, Eloia Emanuelly Dias Silva, Deise Maria Rego Rodrigues Silva, Pedro Henrique Macedo Moura, Pamela Chaves de Jesus, Jessiane Bispo de Souza, Adriana Kelly Santana Correa, Allec Yuri Santos Martins, Anne Gabriela de Freitas Almeida, Lucas Alves da Mota Santana, Lysandro Pinto Borges

**Affiliations:** 1 Division of Phytochemistry and Drug Design, Rajagiri College of Social Sciences (Autonomous), Kalamassery, Kochi, Kerala 683104, India; 2 School of Sciences, Bharata Mata College (Autonomous), Thrikkakara, Kochi, Kerala 682021, India; 3 Department of Pharmacy, Federal University of Sergipe, São Cristóvão 49100-000, Sergipe, Brazil; 4 University of São Paulo, Postgraduate Pharmacy Program (Pathophysiology and Toxicology), São Paulo 05508-000, São Paulo, Brazil; 5 Department of Biology, Federal University of Sergipe, São Cristóvão 49100-000, Sergipe, Brazil; 6 Graduate Program in Dentistry, Federal University of Sergipe, São Cristóvão 49100-000, Sergipe, Brazil

## ⁯⁯

The zoonotic Mpox (formally known as Monkeypox) virus, from the same family as the smallpox virus, is a threat to global public health. This virus mainly affects animals but can be transmitted to humans through contact with infected individuals or animals. Recent global outbreaks and the rapid mutation rate of the virus raise concerns about its potential virulence and transmissibility (Martinez-Fernandez et al., 2023[9]; Imani et al., 2024[7]). Advanced genomic and bioinformatics tools can generate essential information on the evolution of the virus, as well as alerting us to transmission patterns and pathogenic potential (Nnaemeka et al., 2024[14]). This knowledge is essential for developing effective strategies to prevent and control this pathology.

Next-generation bioinformatics tools of artificial intelligence (AI) allow researchers to dissect the intricacies of the Mpox genome. With the weighted gene co-expression network analysis (WGCNA), this enables *in silico* investigation of co-expression networks in various diseases, elucidating the molecular mechanisms of viral diseases and guiding the development of targeted therapies for them (Alarabi et al., 2022[3]). WGCNA and host-pathogen interaction (HPI) analysis helped to explore the complex interplay between the Mpox virus and the human host. WGCNA identified key gene modules significantly impacted by the virus, while HPI analysis revealed how viral proteins manipulate host cellular processes (Tang et al., 2024[16]). The integrated results of the WGCNA and HPI tools highlighted the identification of possible antiviral therapeutic targets, such as kinase inhibitors and hormone-based therapies, which could modulate key signaling pathways such as nuclear factor kappa B (NF-κB), interferons (IFNs), phosphatidylinositol-3 kinase (PI3K), and signal transducer and activator of transcription 3 (STAT3) (Imani et al., 2024[7]). 

MATLAB is another powerful AI platform revolutionizing genomic research, machine learning (ML) and deep learning. AI makes it possible to analyze large data sets efficiently, visualizing complex genomic data. This allows researchers to analyze patterns and understand the pathological mechanism of Mpox and other viruses (MathWorks, 2024[10]). In addition to these tools, Nextclade is a bioinformatics tool vital for Mpox surveillance. Analyzing viral genomes classifies strains into clades (e.g., Clade IIb) and lineages (e.g., B.1), identifying mutations and tracking their evolution. This information aids in understanding genetic diversity and pinpointing the emergence and spread of distinct viral subtypes. While not directly assessing virulence or infectivity, Nextclade indirectly contributes by identifying mutations linked to specific lineages, potentially associated with changes in disease characteristics, such as signs and symptoms. The tool's output seamlessly integrates with Nextstrain for generating phylogenetic trees, visualizing evolutionary relationships, and informing public health strategies by tracking transmission patterns and identifying potential hotspots of viral evolution (Bosmeny et al., 2023[4]; Choi et al., 2024[5]; Aksamentov et al., 2021[2]). In addition, MpoxRadar is a genomic surveillance tool created specifically for Mpox. MpoxRadar allows researchers and public health authorities to obtain information about the disease. It allows rapid analysis of virus variants, even down to mutations, providing information for public health. Its interface is easy to use, with customizable filters and intuitive visualizations, making it possible to access and interpret complex genomic data (Nasri et al., 2023[13]).

Public databases such as NCBI, GISAID and virological.org provide a wealth of viral sequences, while BLAST facilitates in-depth sequence comparisons to identify potentially virulent factors. Parsnp and Muscle allow robust alignments of multiple sequences, revealing patterns of genetic variation between strains. Powerful visualization tools such as Jalview and MEGA reveal evolutionary relationships and potential drug targets. Analyzing the structure of proteins using PDB's web tools, exemplified by the A42R protein (PDB entry 4QWO), makes it possible to identify structural changes that can influence infectivity. Therefore, several existing tools can help in the fight against Mpox (RCSB PDB, 2014[15]). Differential expression analysis using limma and pathway enrichment analysis using GSEA with MSigDB collections (KEGG, WikiPathways, Gene Ontology) reveal virus-host interactions and potential virulence mechanisms. The R statistical software with the corto package allows the creation of informative graphs to effectively communicate these findings (Giorgi et al., 2024[6]).

Rigorous quality control and assembly of raw sequencing data are facilitated by tools like FastQC, Fastp, Flye, and SPADES. Reference-based assembly and variant calling are streamlined using Bowtie2, Pilon, iVar, LoFreq and BCFtools. Variant annotation and impact assessment are achieved with SnpEff and SnpSift. Phylogenetic relationships are elucidated through IQ-Tree 2 and visualized with iTOL. PopArt enables exploration of genetic diversity through halotype networks. MEGA_X_10.0.2 constructs reference sequences for subsequent analyses. Furthermore, tools like SWISS-MODEL facilitate the visualization and comparison of protein conformation models, providing crucial insights into structural variations (Zhu et al., 2023[17]). CheckV v1.0.1, utilizing the checkv-db v1.2 databases, assesses the quality of each Mpox genome assembly by comparing them to reference genomes using a diamond database. This method provides an estimate of genome completeness on a scale of 0-100 %, ensuring the reliability of subsequent analyses by eliminating incomplete sequences (Agarwal et al., 2024[1]). Kraken2 and NCBI SRA human-scrubber remove human reads. Minimap2, BWA-MEM, and Bowtie2 align Illumina unclassified reads to the MPXV genome. PICARD generates duplicate metrics, while Samtools and mosdepth provide coverage metrics. Samtools and iVar generate a consensus sequence. Unicycler produces a hybrid de novo assembly visualized with Bandage and refined with Kraken2. Samtools, BCFtools, and seqtk generate the consensus of this assembly. QUAST compares the consensus sequences against the reference genome (Munoz-Barrera et al., 2023[12]). AGAT, Geneious Prime, and MetaLogo provide valuable insights into functional annotation and the visualization of sequence motifs within specific genomic regions (Monzon et al., 2024[11]).

Advanced bioinformatics techniques are crucial for understanding the Mpox virus and developing effective control strategies. By analyzing the virus's genome, researchers can gain insights into its evolution, transmission patterns, and virulence. Tools like WGCNA, HPI analysis, and MATLAB enable the identification of key genes, host-pathogen interactions, and potential therapeutic targets. Nextclade facilitates viral classification and mutation tracking, aiding in surveillance efforts. A wide array of software tools, from sequence alignment and phylogenetic analysis to protein structure prediction and differential expression analysis, empowers researchers to comprehensively investigate the Mpox virulence and develop effective strategies for containment and mitigation of this virus. 

This integrated approach using AI has led to the development of regulatory networks involving transcription factors and protein kinases, making it possible to understand the mechanisms responsible for viral evasion of the immune system and disease progression (Loganathan et al., 2024[8]). With the AI tool and ML algorithms, it is possible to predict viral mutations, model evolutionary pathways and assess the impact of these changes on transmissibility. This is important for responding to emerging infectious diseases such as Mpox, as it allows for the development of effective and agile targeted interventions, minimizing the impacts of these endemics. This integration of AI and ML presents an important advance in the understanding of Mpox and other infectious diseases, making MATLAB an essential tool for future scientific research (Nnaemeka et al., 2024[14]). In conclusion, the application of advanced AI techniques is instrumental in unraveling the complexities of the Mpox virus. By leveraging advanced sequencing analyses using these tools to unravel the genetic intricacies of Mpox, we can pave the way for optimized research, enabling more effective strategies for containment and mitigation.

## Notes

Marina dos Santos Barreto and Lysandro Pinto Borges (Department of Pharmacy, Federal University of Sergipe, São Cristóvão 49100-000, Sergipe, Brazil; E-mail: lysandro.borges@gmail.com) contributed equally as corresponding author.

## Declaration

### Conflict of interest

The authors declare no conflicts of interest related to this research. 

### Using artificial intelligence (AI)

AI was used for writing assistance in this manuscript. The authors take full responsibility for the content.
